# Design and Characterization of a New Phenoxypyridine–Bipyridine-Based Tetradentate Pt(II) Complex Toward Stable Blue Phosphorescent Emitters

**DOI:** 10.3390/molecules31020373

**Published:** 2026-01-20

**Authors:** Da-Gyung Lim, Ju-Hee Lim, Chan Hee Ryu, Kang Mun Lee, Youngjin Kang

**Affiliations:** 1 Division of Science Education, Kangwon National University, Chuncheon 24341, Gangwon, Republic of Korea; ldkland@naver.com (D.-G.L.); limjuhee6948@naver.com (J.-H.L.); 2 Department of Chemistry, Institute for Molecular Science and Fusion Technology, Kangwon National University, Chuncheon 24341, Gangwon, Republic of Korea

**Keywords:** tetradentate–platinum(II)-complex, phosphorescence, phenoxy–pyridine framework, bipyridine-derived ligand

## Abstract

Although various phosphorescent organic light-emitting diodes (PhOLEDs) have been developed, their lifetimes remain shorter than those of fluorescent OLEDs. In this study, a novel Pt(II) complex featuring a tetradentate ligand composed of bipyridine and phenoxypyridine, referred to as **LL-O**, was synthesized and fully characterized to evaluate its potential as a dopant for PhOLEDs. Geometry-optimized calculations indicate that **LL-O** adopts a distorted square–planar structure around the Pt(II) center. The complex displays bluish-green emission with maxima at 490 and 518 nm. However, it exhibits a low photoluminescence quantum yield (4%), primarily due to a dominant non-radiative decay rate that surpasses the radiative decay rate. Natural transition orbital analysis reveals that the emission of **LL-O** originates from a combination of triplet ligand-centered (^3^LC), triplet ligand-to-ligand charge-transfer (^3^LL′CT), and triplet metal-to-ligand charge-transfer (^3^MLCT) transitions. This compound also demonstrates high thermal stability (decomposition temperature > 340 °C) and an appropriate HOMO energy level (−5.58 eV), making it suitable for use as a dopant in versatile PhOLEDs.

## 1. Introduction

To fabricate organic light-emitting diodes (OLEDs) with low power consumption and high efficiency, it is essential to develop blue phosphorescent materials [1,2,3,4,5]. Numerous studies have investigated ways to improve the efficiency of OLEDs. These efforts have resulted in the development of phosphorescent materials with superior performance to that of their fluorescent counterparts [6,7,8,9,10,11]. However, fluorescent materials are still preferred over phosphorescent materials for blue emission in commercial applications due to the relatively short lifetimes of the latter [12,13,14,15]. Phosphorescence relies on intersystem crossing (ISC), wherein singlet excitons are converted into triplet excitons. To enhance the efficiency of ISC, the heavy atom effect is utilized by incorporating heavy atoms into the molecular structure. Ir and Pt are widely employed as heavy metals in emissive materials [16,17,18]. While Ir(III) complexes show excellent efficiency, they typically possess broad emission spectra [19,20]. In contrast, Pt(II) complexes not only exhibit high efficiency but also have narrow emission bandwidths, resulting in improved color purity for blue phosphorescent dopants. Therefore, Pt(II) complexes are considered more suitable for application in organic display devices [21,22].

Selection of ligands is critical for the development of blue phosphorescent Pt materials. This is because electronic transitions in Pt-based phosphorescent complexes are known to primarily arise from the local excitation (LE) of the ligand along with metal-to-ligand charge transfer (MLCT) transitions [21]. Therefore, while designing ligands, careful consideration must be given to selecting ligands that either possess high triplet energy levels or are capable of generating a strong ligand field upon coordination with Pt. Tetradentate ligand architectures are particularly attractive for blue phosphorescent Pt(II) complexes, as they provide enhanced kinetic and thermal stability by suppressing ligand dissociation and structural rearrangement. The rigid coordination framework also restricts large-amplitude excited-state structural relaxation, which is a key factor in mitigating non-radiative decay processes in high-energy blue emitters. Moreover, tetradentate chelation enables more precise control over the balance between ligand-centered and charge-transfer excited states, allowing for fine tuning of emission energy and photophysical properties. With these considerations in mind, many studies have reported novel tetradentate ligands based on various combinations of high-triplet-energy moieties such as pyrazole [23,24,25,26], carbazole [27,28,29], triazole [30,31,32,33], carbene [34,35,36,37,38], and bipyridine [39,40]. The ligand that particularly drew our attention was phenoxypyridine. This ligand demonstrates near-unity quantum efficiency when coordinated with Pt and exhibits phosphorescence in the blue region [41]. However, despite these promising properties, it has not yet been extensively studied [42].

Our research group has been continuously studying bipyridine-based ligands, which show high triplet energy and high quantum efficiency when coordinated with Pt [43] or Ir [44]. Hence, we hypothesize that the development of a tetradentate ligand combining bipyridine and phenoxypyridine would afford promising Pt complexes as potential candidates for blue phosphorescent emitters. In this study, structural characteristics were analyzed by ^1^H and ^13^C NMR spectroscopy, photophysical properties by UV-Vis absorption and photoluminescence (PL) spectroscopy, electrochemical behavior by cyclic voltammetry (CV), and thermal stability by thermogravimetric analysis (TGA) in order to evaluate the potential of a new compound as an OLED triplet emitter.

## 2. Results and Discussion

### 2.1. Synthesis and Characterization of the Pt(II) Complex

**Scheme 1:**
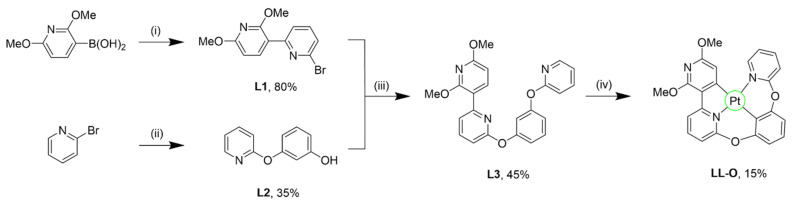
Synthesis procedure for the tetradentate-ligand-based Pt(II) complex **LL-O**. Reagent and conditions: (i) 2,6-dibromopyridine, Pd(PPh_3_)_4_, K_2_CO_3_, THF/H_2_O (3/2, *v*/*v*), 80 °C, 18 h. (ii) 1,3-benzenediol, 1-methylimidazole, CuI, K_2_CO_3_, toluene/pyridine (1/1, *v*/*v*), 120 °C, 48 h. (iii) CuI, picolinic acid, K_3_PO_4_, DMSO, 110 °C, 72 h. (iv) Bis(benzonitrile)dichloroplatinum(II), *p*-xylene, 110 °C, 24 h.

To synthesize complex **LL-O** (Scheme 1), the bipyridine derivative **L1** was first prepared using a Suzuki coupling reaction. Subsequently, the phenoxypyridine derivative **L2** was synthesized via an Ullmann coupling process. The coupling of **L1** and **L2** under similar Ullmann conditions afforded the tetradentate ligand **L3** (45% yield), thereby completing the ligand framework. Finally, **L3** was treated with 1 equiv. of bis(benzonitrile)dichloroplatinum(II) to produce a square–planar Pt(II) complex **LL-O**, featuring a tetradentate coordination environment, in 15% yield. Overall, this synthetic route demonstrates the successful construction of a robust tetradentate coordination system through sequential cross-coupling reactions, followed by complexation with a Pt(II) precursor. The moderate ligand yield and relatively lower metalation yield suggest steric and electronic influences arising from the phenoxy and bipyridyl moieties, which may affect the coordination efficiency during complex formation.

The Pt(II) complex was thoroughly characterized using ^1^H and ^13^C NMR spectroscopy (Figures S1–S3). In the ^1^H NMR spectrum, a distinct singlet was observed at 3.9–4.1 ppm, corresponding to the methoxy protons. Although partially overlapped with the dichloromethane-*d*_2_ solvent peak, the ^13^C NMR spectrum exhibits a characteristic resonance near 53 ppm, further confirming the presence of two methoxy groups in the complex (Figure S3).

In addition, high-resolution mass spectrometry (HRMS) analysis shows a molecular ion peak that is in excellent agreement with the calculated molecular mass of **LL-O** (Figure S4), providing further confirmation of the successful formation of the target Pt(II) complex. Elemental analysis (C, H, N) also closely matches the theoretical values, supporting the high purity and correct molecular composition of the synthesized compound.

**Figure 1:**
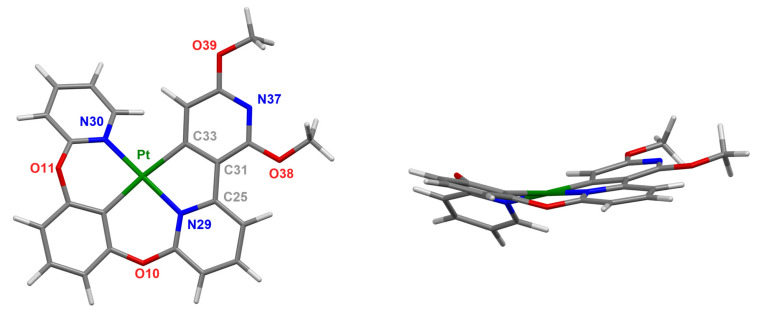
(**left**) Top view and (**right**) side view of ground-state (S_0_)-optimized structures of **LL-O** derived through density functional theory calculations.

Furthermore, the ground-state (S_0_) geometry of the **LL-O** complex was optimized using density functional theory (DFT) calculations (Figure 1). The optimized structure revealed a pronounced distortion of the terminal pyridine ring in the coordinated ligand around the central Pt(II) atom (Figure 1, side view). This distortion is attributed to torsional strain and an asymmetric coordination environment within the ligand framework, which perturb the ideal square–planar geometry of the complex. The sum of the bond angles around the Pt(II) center was calculated to be 361.39°, which is close to an ideal square–planar geometry. While this value alone does not quantitatively describe out-of-plane distortion, the side-view molecular structure reveals a subtle but distinct nonplanarity of the ligand framework. In particular, the C33–C31–C25–N29 torsional angle was calculated to be approximately 5°, indicating a slight twisting of the pyridine-based ligand coordinating the Pt(II) center. These findings suggest that the rigid tetradentate coordination framework restricts intramolecular degrees of freedom, thereby suppressing structural fluctuation and vibrational motion of the Pt(II) core. At the same time, the nonplanar molecular geometry hinders efficient intermolecular packing, which in turn weakens intermolecular interactions and suppresses excimer emission in the solid state.

### 2.2. Photophysical Properties of the ***LL-O*** Complex

**Figure 2:**
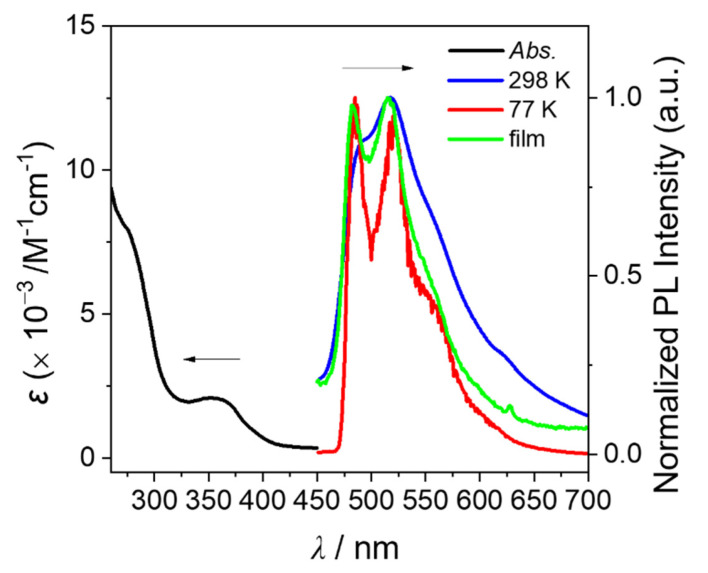
(left) UV−Vis absorption and (right) PL spectra of **LL-O** (*λ*_ex_ = 382 nm). Black line: absorption spectra in dichloromethane (1.0 × 10^−4^ M); blue and red lines: PL spectra in dichloromethane (1.0 × 10^−4^ M) at 298 and 77 K, respectively; green line: PL spectra of PMMA film doped with 5 wt.% **LL-O** at 298 K.

**Figure 3:**
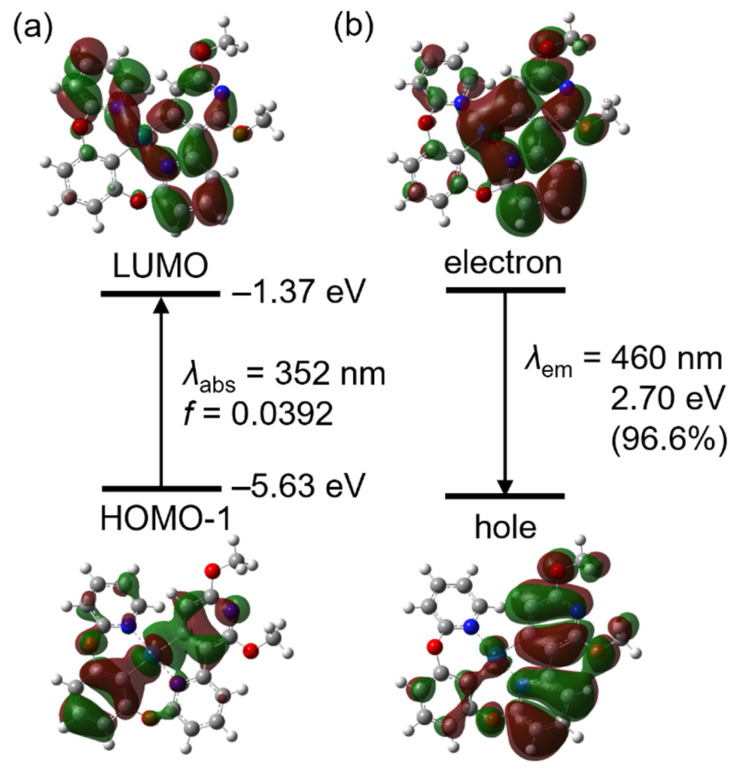
(**a**) Frontier molecular orbitals for **LL-O** at their ground state (S_0_) with their relative energies from DFT calculation (isovalue 0.02). The transition energy (in nm) was calculated using the TD-PBE0/GENCP method with 6-31G(d,p) basis sets. (**b**) The natural transition orbitals (NTOs) of the low-lying excited states for **LL-O**: hole and electron distributions for the T_1_ state based on the optimized S_0_-geometry.

The optical properties of **LL-O** were examined by ultraviolet–visible (UV–vis) absorption and PL measurements in degassed dichloromethane solution (Figure 2 and Table 1). A strong low-energy absorption band was observed around 350 nm (ε ≈ 21,000 M^−1^·cm^−1^), which is primarily attributed to ^1^MLCT (metal-to-ligand charge transfer) and ^1^LL′CT (ligand-to-ligand charge transfer) transitions. To gain deeper insights into the origin of these absorptive transitions, time-dependent density functional theory (TD-DFT) calculations were performed based on the S_0_-optimized geometry of the complex (Figure 3). The computational analysis revealed that the main electronic excitations arise from transitions between the highest occupied molecular orbitals (HOMO) and HOMO-1 and the lowest unoccupied molecular orbital (LUMO), confirming that the observed absorption band predominantly originates from the charge-transfer (CT) character within the complex.

Notably, the electron density distributions of the HOMO and HOMO-1 orbitals were mainly localized over the methoxy–pyridine and phenyl segments (64% and 37%, respectively), with an additional contribution from the Pt(II) center (~34%). In contrast, the LUMO was predominantly confined to the terminal pyridine moiety (~69%), while the contribution from the metal center was minimal (~4%). These results suggest that low-energy electronic transitions of the Pt(II) complex primarily originate from ^1^MLCT (metal-to-ligand charge transfer) and ^1^LL′CT (ligand-to-ligand charge transfer) characteristics. These results are consistent with those reported previously for the Pt[N^C-O-popy] compound [41].

The **LL-O** complex exhibited bluish-green phosphorescence in degassed dichloromethane solution at 298 K, with well-defined emission maxima at 490 and 518 nm (Figure 2). The emission spectrum displayed a well-structured profile, characteristic of CT-dominated phosphorescence. This complex exhibited emission behavior similar to that of PtpypyOczpy, previously reported by our group [45], which can be ascribed to the comparable T_1_ energy levels of the pyridyl–carbazole and phenoxypyridine frameworks. The structural resemblance between these two ligand systems results in analogous photophysical characteristics, reflecting their similar triplet-state energetics.

To gain further insights into the emissive transitions of the complex, natural transition orbital (NTO) calculations were performed for the lowest-energy triplet state (T_1_) based on the ground-state (S_0_) optimized geometry. As shown in Figure 3b, both electron and hole densities were primarily localized on the methoxy–pyridine moiety and the adjacent pyridine unit, with partial delocalization of the electron density extending toward the terminal pyridine fragment and the Pt(II) center. These results suggest that the emission of **LL-O** originates from a combination of ^3^LC, ^3^LL′CT, and ^3^MLCT, among which the ^3^LC and ^3^LL′CT components make the maximum contribution to the overall phosphorescent process.

The PL spectra of **LL-O** were measured in degassed dichloromethane solution at 77 K and in a PMMA matrix (5 wt.%) to investigate its emission behavior under rigid conditions (Table 1). Compared with the spectrum obtained in the room-temperature solution, it exhibited a distinctly blue-shifted emission under both the aforementioned rigid environments. This shift toward shorter wavelengths can be attributed to the suppression of molecular motions in the solid or frozen states, which limits structural relaxation in the triplet excited state and consequently hinders the stabilization of the low-energy emissive state, leading to higher-energy emission.

In the PMMA matrix (5 wt.%), the absolute PL quantum yield (Φ_em_) of **LL-O** was measured to be 4%, indicating a relatively low emissive efficiency (Table 1). The observed emission lifetime (τ_obs_) was 9.06 μs (Table 1 and Figure S6), which confirmed the presence of slow phosphorescent radiative decay. Based on the Φ_em_ and τ_obs_ values, the radiative (k*_r_*) and non-radiative (k*_nr_*) decay rate constants were calculated to be 4.42 × 10^3^ s^−1^ and 1.06 × 10^5^ s^−1^, respectively, indicating that the non-radiative decay rate overwhelmingly dominates over the radiative process. The relatively long observed phosphorescence lifetime of **LL-O** therefore reflects a small radiative decay rate in the presence of a highly efficient non-radiative deactivation pathway, which accounts for its low photoluminescence efficiency.

The electrochemical properties of **LL-O** were investigated by CV to determine the energy levels of the complex. The HOMO and LUMO energy levels, estimated from the onset oxidation and reduction potentials, were calculated to be −5.58 and −2.54 eV, respectively (Table 1 and Figure S5). Thermal stability was further examined by TGA (Table 1 and Figure S7). The temperature corresponding to a 5% weight loss (Td_5_) was recorded as 340 °C, which confirmed that **LL-O** exhibits excellent thermal stability consistent with the inherent robustness of tetradentate Pt(II) complexes.

## 3. Materials and Methods

### 3.1. General Considerations

All operations were performed under an inert nitrogen atmosphere using standard Schlenk and glove box techniques. Anhydrous grade solvents (tetrahydrofuran (THF), toluene, dimethyl sulfoxide (DMSO), and *p*-xylene) were dried by passing them through an activated alumina column and storing over activated molecular sieves (5 Å). Spectrophotometric-grade solvent (dichloromethane) was used as received from Alfa Aesar (Haverhill, MA, USA). All commercial reagents were purchased from Sigma-Aldrich (St. Louis, MO, USA) or Alfa Aesar and used as received. Deuterated solvents (chloroform-*d* (CDCl_3_) and methylene chloride-*d*_2_ (CD_2_Cl_2_)) were obtained from Cambridge Isotope Laboratories and used after drying over activated molecular sieves (5 Å). NMR spectra were recorded on a JEOL 400 MHz spectrometer (JEOL Ltd., Tokyo, Japan) (400.13 MHz for ^1^H and 100.62 MHz for ^13^C). Chemical shifts were indicated in ppm. High-resolution mass spectrometry (HRMS) measurements were performed using a JMS-T2000GC mass spectrometer (JEOL Ltd., Tokyo, Japan). Elemental analyses (C, H, N) were carried out using an EA3000 elemental analyzer (Eurovector S.p.A., Pavia, Italy). TGA was performed under a N_2_ atmosphere using TA Instrument Thermogravimetric Analyzer Q500 (TA Instruments, New Castle, DE, USA) at a heating rate of 10 °C·min^−1^ from 25 to 800 °C. CV measurements were performed on a PGSTAT12 system (Metrohm Autolab B.V., Utrecht, Netherlands) using a three-electrode cell, with Pt working and counter electrodes and a reference electrode comprising 0.1 M Ag/AgNO_3_ in acetonitrile at 25 °C (supporting electrolyte: 0.1 M solution of tetrabutylammonium hexafluorophosphate in dichloromethane). The oxidation and reduction potentials of **LL-O** were recorded at a scan rate of 100 mV s^−1^ and were reported with reference to the ferrocene/ferrocenium (Fc/Fc^+^) redox couple.

### 3.2. Synthesis of 6-Bromo-2′,6′-dimethoxy-2,3′-bipyridine, ***L1***

Compound **L1** was prepared according to a literature reported procedure [45].

### 3.3. Synthesis of 3-(Pyridin-2-yloxy)phenol, ***L2***

To prepare **L2**, a dry pressure vessel was first charged with 2-bromopyridine (3.17 mL, 33.30 mmol), resorcinol (5.50 g, 49.96 mmol), 1-methylimidazole (1.33 mL, 16.69 mmol), K_2_CO_3_ (9.21 g, 66.64 mmol), pyridine (20 mL), and toluene (20 mL). The mixture was degassed for 10 min, after which CuI (0.63 g, 3.31 mmol) was added, followed by an additional 10 min of degassing. The vessel was then sealed and heated at 120 °C for more than 2 days. After cooling to room temperature, the reaction mixture was filtered through a glass frit, and the filtrate was collected using toluene as the transfer solvent. (If solid residues remained, they were rinsed sequentially with toluene, 3% aqueous acetic acid, and EtOH, and combined with the filtrate.) The combined organic layers were dried over anhydrous MgSO_4_, filtered, and concentrated under reduced pressure. The crude material was recrystallized from toluene to afford the product as a dark gray solid in 35% yield. ^1^H NMR (400 MHz, chloroform-*d*) δ 8.21 (dd, *J* = 5.0, 2.0 Hz, 1H), 7.72–7.67 (m, 1H), 7.23 (d, *J* = 8.1 Hz, 1H), 7.01 (dd, *J* = 7.3, 4.9 Hz, 1H), 6.94–6.90 (m, 1H), 6.72–6.63 (m, 2H), 6.61 (t, *J* = 2.4 Hz, 1H), 5.58 (s, 1H).

### 3.4. Synthesis of 2′,6′-Dimethoxy-6-(3-(pyridin-2-yloxy)phenoxy)-2,3′-bipyridine, ***L3***

To obtain **L3**, a Schlenk flask was first charged with **L1** (1.434 g, 4.61 mmol), **L2** (0.718 g, 3.84 mmol), CuI (0.073 g, 0.384 mmol), picolinic acid (0.094 g, 0.768 mmol), and K_3_PO_4_ (1.63 g, 7.68 mmol), and then evacuated under vacuum. Next, DMSO (15 mL) was added to the flask, and the mixture was heated and stirred at 95–105 °C for 3 days under a nitrogen atmosphere. After cooling to room temperature, the reaction mixture was diluted with ethyl acetate (EA) and filtered through Celite on a glass frit. The filtrate was extracted with EA and distilled water. The combined organic layers were dried over anhydrous Na_2_SO_4_, filtered, and concentrated under reduced pressure. The crude material was purified by silica gel column chromatography using EA and hexane in a ratio of 1:5 as the eluent. The product was obtained as a white solid in 45% yield. ^1^H NMR (400 MHz, chloroform-*d*) δ 8.26–8.18 (m, 2H), 7.81 (d, *J* = 7.8 Hz, 1H), 7.71–7.62 (m, 2H), 7.39 (t, *J* = 8.1 Hz, 1H), 7.05–6.96 (m, 4H), 6.93–6.87 (m, 1H), 6.76 (dd, *J* = 8.0, 0.9 Hz, 1H), 6.35 (d, *J* = 8.3 Hz, 1H), 4.04 (s, 3H), 3.95 (s, 3H).

### 3.5. Synthesis of ***LL-O***

A Schlenk flask was charged with **L3** (0.50 g, 0.841 mmol), bis(benzonitrile)dichloroplatinum(II) (0.40 g, 0.847 mmol), and *p*-xylene (5 mL). The reaction mixture was heated and stirred at 110 °C for 1 day under a nitrogen atmosphere. After cooling to room temperature, the mixture was washed with dichloromethane and concentrated under reduced pressure. The crude product was purified by silica gel column chromatography using dichloromethane as the eluent, affording the product as a yellow solid in 15% yield. ^1^H NMR (400 MHz, methylene chloride-*d*_2_) δ 9.09 (dd, *J* = 6.1, 1.8 Hz, 1H), 8.30 (dd, *J* = 8.0, 1.3 Hz, 1H), 7.96 (ddd, *J* = 8.6, 7.1, 1.8 Hz, 1H), 7.88 (td, *J* = 8.0, 1.2 Hz, 1H), 7.34 (dd, *J* = 8.4, 1.5 Hz, 1H), 7.17–7.13 (m, 2H), 7.10–7.01 (m, 3H), 6.45 (d, *J* = 1.2 Hz, 1H), 4.09 (s, 3H), 3.93 (s, 3H). ^13^C NMR (101 MHz, methylene chloride-*d*_2_) δ 188.16, 166.34, 162.94, 159.95, 159.55, 156.90, 155.03, 154.46, 153.08, 140.81, 140.19, 126.00, 122.94, 120.53, 116.50, 116.21, 111.97, 110.88, 110.34, 106.53, 54.32, 53.49. Anal. calcd for C_23_H_17_N_3_O_4_Pt: C 46.47, H 2.88, N 7.07; found C 46.43, H 2.85, N 7.10.

### 3.6. UV/Vis Absorption and PL Measurements

Agilent VARIAN Cary 100 Conc UV–vis spectrophotometer (Agilent Technologies, Santa Clara, CA, USA) and HORIBA Fluoromax-4P luminescence spectrophotometer (HORIBA Scientific, Kyoto, Japan) were used to obtain UV-vis absorption and PL spectra, respectively. The UV-vis absorption data of the solution were measured using degassed dichloromethane in a 1 cm quartz cuvette (1.0 × 10^−4^ M). PL measurements were collected using degassed dichloromethane (1.0 × 10^−4^ M) at ambient conditions and 77 K. PL data in the film state (5 wt.% doped in PMMA) was also collected on a 10 × 10 mm quartz plate (thickness = 1 mm). The absolute PLQYs of the film were measured at 298 K using a Quantaurus-QY Absolute PL Quantum Yield Spectrometer (C11347, Hamamatsu Photonics K.K., Hamamatsu, Japan). Phosphorescence decay lifetimes in the film were measured at 298 K using an FS5 spectrometer (Edinburgh Instruments Ltd., Livingston, UK) equipped with an EPLED picosecond pulsed LED source (375 nm) coupled with multi-channel scaling (MCS). Lifetimes were analyzed by double-exponential tail-fitting.

### 3.7. Theoretical Calculations

The geometries of the Pt(II) complex in their S_0_ state were fully optimized at the PBE0 level. TD-DFT [46] was used to investigate electronic transition energies. Solvent effects were evaluated using a self-consistent reaction field method based on the integral equation formalism of the polarizable continuum model with dichloromethane as the solvent [47]. The LANL2DZ and 6-31G(d,p) basis sets were used for the Pt atom and nonmetal atoms, respectively. The origin of excited singlet and triplet states, including the vertical excitation orbitals and NTOs, were calculated using TD-DFT at the same level. All calculations were conducted using GAUSSIAN 16 software [48]. The percentage contribution of a group in a molecule to each molecular orbital was calculated using the GaussSum 3.0 program [49], and visualizations were performed using GaussView 6 [50].

## 4. Conclusions

In summary, a new tetradentate Pt(II) complex, **LL-O**, integrating bipyridine and phenoxy–pyridine moieties, was successfully synthesized and fully characterized. The complex exhibits bluish-green phosphorescence with well-structured emission bands at 490 and 518 nm in solution. Electronic-structure and natural transition orbital analyses revealed that the emission originates from mixed ^3^LC, ^3^LL′CT, and ^3^MLCT transitions, with the ^3^LC component being the most dominant contributor to the emissive state. The relatively long phosphorescence lifetime (τ_obs_ = 9.06 μs) and low photoluminescence quantum yield (Φ_em_ = 4%) indicate that non-radiative decay pathways are more prevalent than radiative ones. Electrochemical and thermal studies further demonstrated that **LL-O** possesses appropriate frontier orbital energy levels (HOMO: −5.58 eV, LUMO: −2.54 eV) and excellent thermal stability (Td_5_ = 340 °C), consistent with the inherent robustness of tetradentate Pt(II) frameworks. Overall, this study highlights that combining bipyridine and phenoxy–pyridine units provide a chemically stable coordination environment capable of sustaining mixed charge-transfer and ligand-centered transitions. Future work will focus on tuning the ligand field and modifying substituents to suppress non-radiative deactivation and enhance the photophysical efficiency of Pt(II) complexes.

## Figures and Tables

**Table 1:** Optical and thermal properties of the tetradentate Pt(II) complex **LL-O**.

Compound	*λ*_abs_ ^1^/nm (ε × 10^−3^ M^−1^ cm^−1^)		*λ*_ex_ ^1^ /nm	*λ*_em_ ^1^/nm		*λ*_em_ ^2^/nm	FWHM ^3^ /nm
				298 K	77 K	Film	
**LL-O**	358 (2.1), 275 (8.0)		382	490, 518	485, 517	483, 515	103
	**Φ_em_** **^2,4^**	**HOMO ^5^** **/eV**	**LUMO ^6^** **/eV**	**τ_obs_ ^2^** **/** **μ** **s**	***k*** **_r_ ^7^** **/** **×** **10^3^ s^−1^**	***k*** **_nr_ ^8^** **/** **×** **10^5^ s^−1^**	**Td_5_ ^9^** **/°C**
	0.04	−5.58	−2.54	9.06	4.42	1.06	340

^1^ Concentration = 1.0 × 10^−4^ M in dichloromethane. ^2^ Measured using spin-coated PMMA film doped with each Pt(II) compound (5 wt.%). ^3^ Full-width at half-maximum of the PL spectrum obtained in dichloromethane at 298 K. ^4^ Absolute PL quantum yield. Calculated from the ^5^ oxidation or ^6^ reduction onset potential vs. ferrocene/ferrocenium^+^ in CV. ^7^ Radiative decay constant *k*_r_ = Φ_em/_τ_obs_. ^8^ Non-radiative decay constant *k*_nr_ = *k*_r_(1/Φ_em_ − 1). ^9^ 5 wt.% decomposition temperature (5% loss).

## Data Availability

Data generated or analyzed during this study are provided in full within the published article and its Supplementary Materials.

## Supplementary Materials

The following supporting information can be downloaded at: https://www.mdpi.com/article/10.3390/molecules31020373/s1, Figure S1: ^1^H NMR spectra of **L2** in CDCl_3_ (* from residual CHCl_3_ in CDCl_3_); Figure S2: ^1^H NMR spectra of **L3** in CDCl_3_ (* from residual CHCl_3_ in CDCl_3_); Figure S3: ^1^H (top) and ^13^C (bottom) NMR spectra of **LL-O** in CD_2_Cl_2_ (* from residual CH_2_Cl_2_ in CD_2_Cl_2_); Figure S4: High-resolution mass spectrum (HRMS) of **LL-O**; Figure S5: Consecutive cyclic voltammogram (CV) curves for **LL-O** showing (a) reduction and (b) oxidation (5 cycles, 0.5 mM in dichloromethane, scan rate = 100 mV/s); Figure S6: Emission decay curve obtained at each emission maximum for **LL-O** in the PMMA film states. The red-line corresponds to the double-exponential fitting curve for the experimental curve; Figure S7: Thermogravimetric analysis curves of **LL-O**; Figure S8: The selected frontier orbitals of **LL-O** from PBE0 calculations (Isovalue = 0.02 a.u.) at the ground state (S_0_) optimized geometries in dichloromethane; Table S1: Computed absorption wavelengths (*λ*_calc_) and oscillator strengths (*f*_calc._) for **LL-O** in its ground state (S_0_) optimized geometry in dichloromethane. The calculations were performed using the TD-PBE0 /GENCP method; Table S2: Molecular orbital energies (E, eV) and molecular orbital distributions (%) of **LL-O** for the ground-state (S_0_) optimized geometry in dichloromethane; Table S3: Cartesian coordinates of the ground state (S_0_) fully optimized geometry of **LL-O** from PBE0 calculations in dichloromethane (in Å).
