# Supplementary material for: Design and Characterization of a New Phenoxypyridine–Bipyridine-Based Tetradentate Pt(II) Complex Toward Stable Blue Phosphorescent Emitters
Source: Molecules. 2026 Jan 20;31(2):373. doi: 10.3390/molecules31020373 (PMC12844317; doi:10.3390/molecules31020373)
Supplement: Supplementary file 1 [file molecules-31-00373-s001.zip › molecules-4040307-supplementary.pdf]

Supporting Information

Design and Characterization of a New Phenoxypyridine–Bipyridine-Based Tetradentate Pt(II) Complex Toward Stable Blue Phosphorescent Emitters

Da-Gyung Lim [1,†], Ju-Hee Lim [1,†], Chan Hee Ryu [2,*] , Kang Mun Lee [2,*] and Youngjin Kang [1,*]

[1] Division of Science Education, Kangwon National University, Chuncheon 24341, Gangwon, Republic of Korea; ldkland@naver.com (D.-G.L.); limjuhee6948@naver.com (J.-H.L.)

[2] Department of Chemistry, Institute for Molecular Science and Fusion Technology, Kangwon National University, Chuncheon 24341, Gangwon, Republic of Korea

***** Correspondence: cksgml2300@kangwon.ac.kr (C.H.R.); kangmunlee@kangwon.ac.kr (K.M.L.); kangy@kangwon.ac.kr (Y.K.)

[†] These authors contributed equally to this work.

Contents

^{1}H and ^{13}C NMR spectra for Pt(II) complex and their precursors ······························ S2−S3

High-resolution mass spectrum (HRMS) for **LL-O** ··· S4

Consecutive cyclic voltammogram curves for **LL-O** ··· S5

Emission decay curves of **LL-O** in PMMA film states ·· S5

Thermogravimetric analysis curve of **LL-O** ·· S6

Theoretical calculation results for **LL-O** ·· S7−S8

Cartesian coordinates of optimized geometries for **LL-O** in dichloromethane ··············· S9

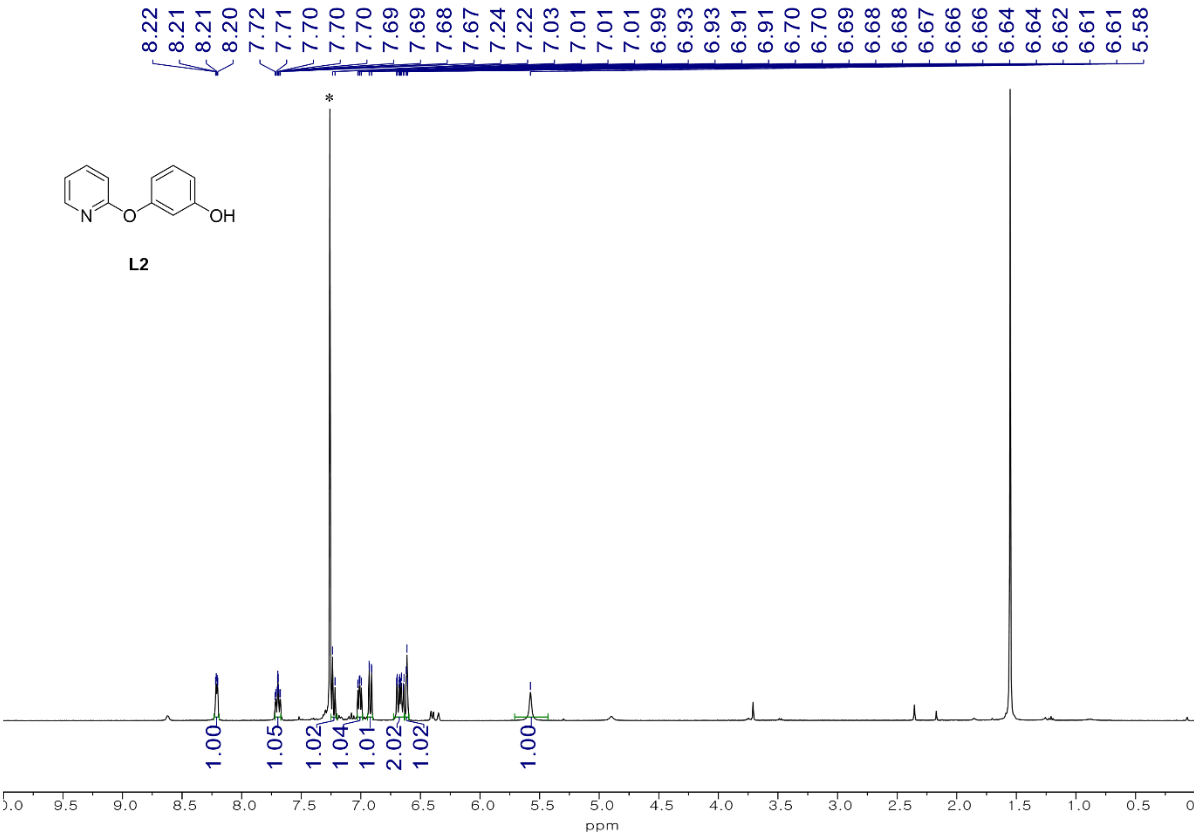

Figure S1. ^{1}H NMR spectra of **L2** in $CDCl_3$ (* from residual $CHCl_3$ in $CDCl_3$).

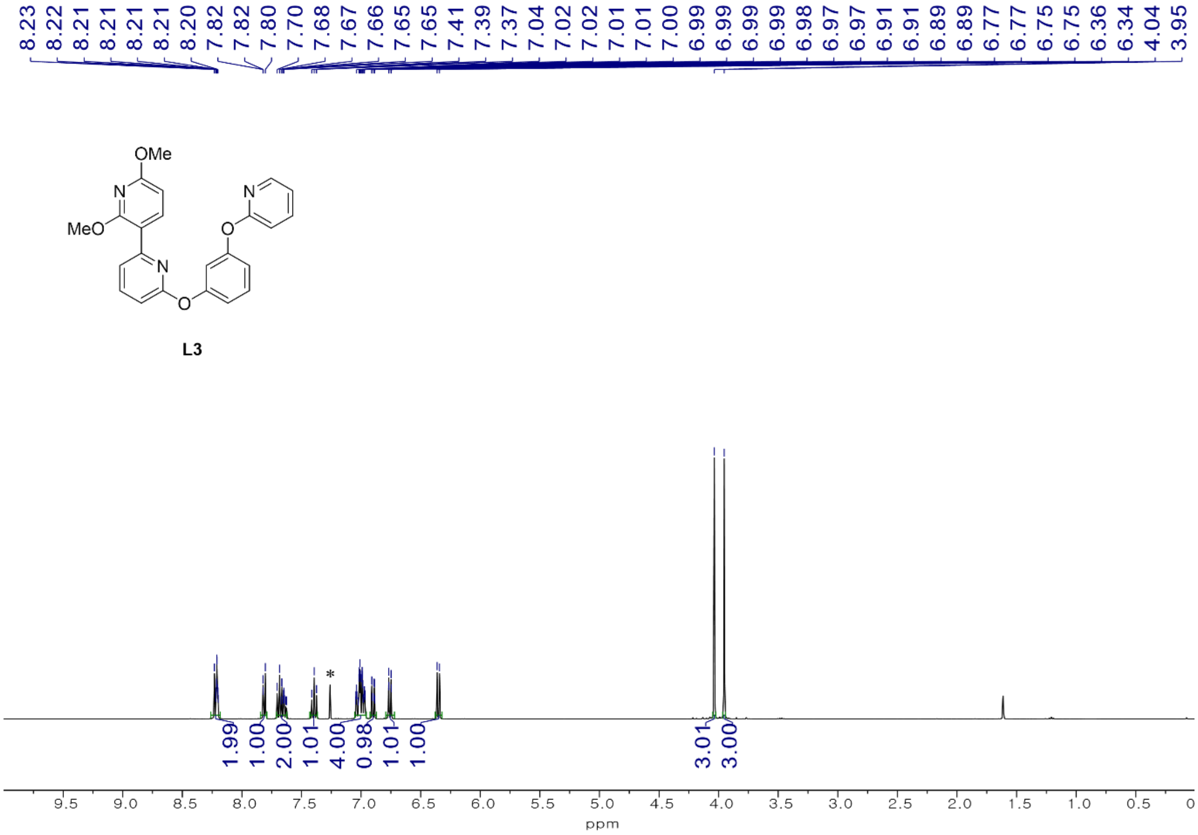

Figure S2. ^{1}H NMR spectra of **L3** in $CDCl_3$ (* from residual $CHCl_3$ in $CDCl_3$).

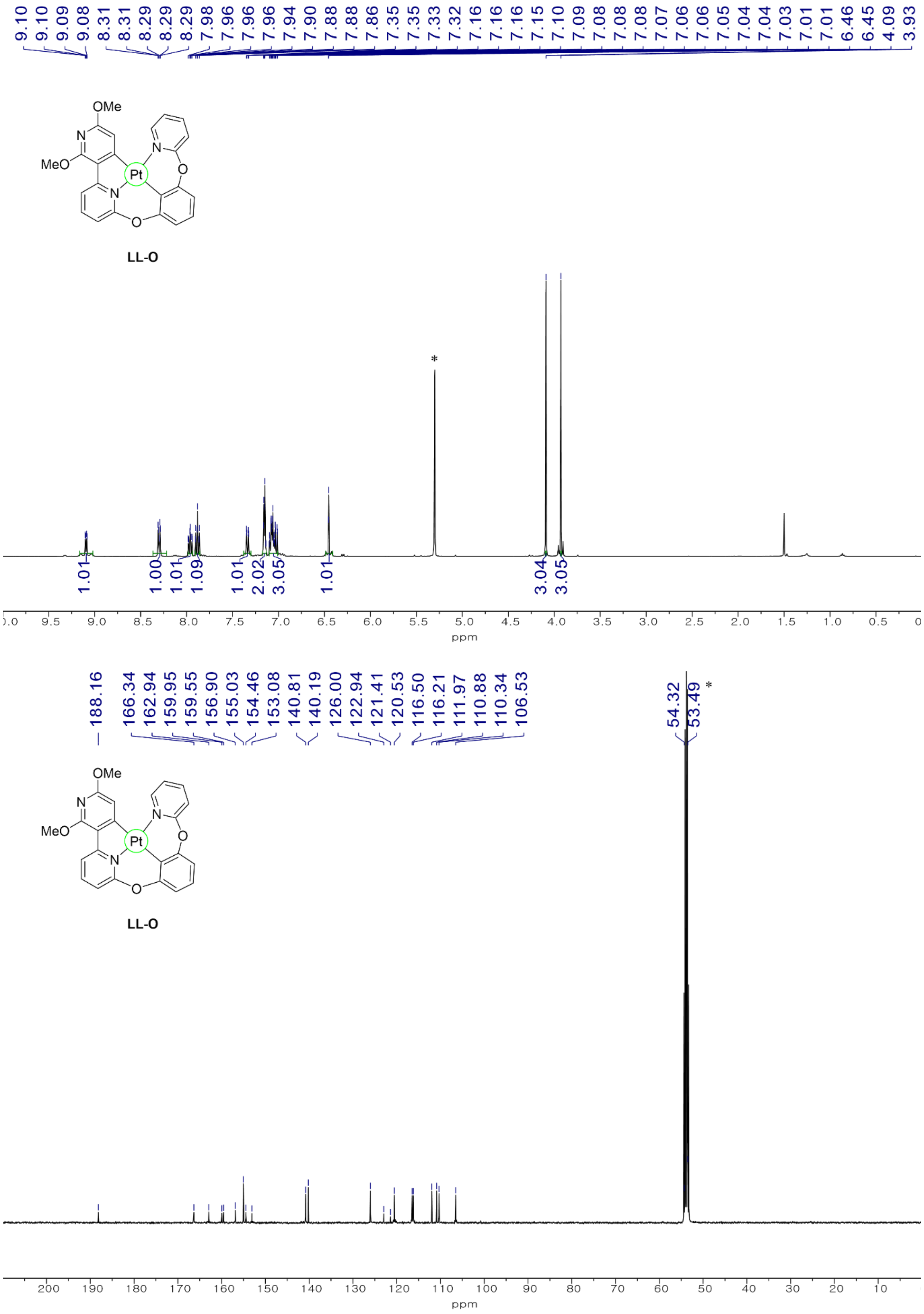

Figure S3. ^{1}H (top) and ^{13}C (bottom) NMR spectra of **LL-O** in CD_2Cl_2 (* from residual CH_2Cl_2 in CD_2Cl_2).

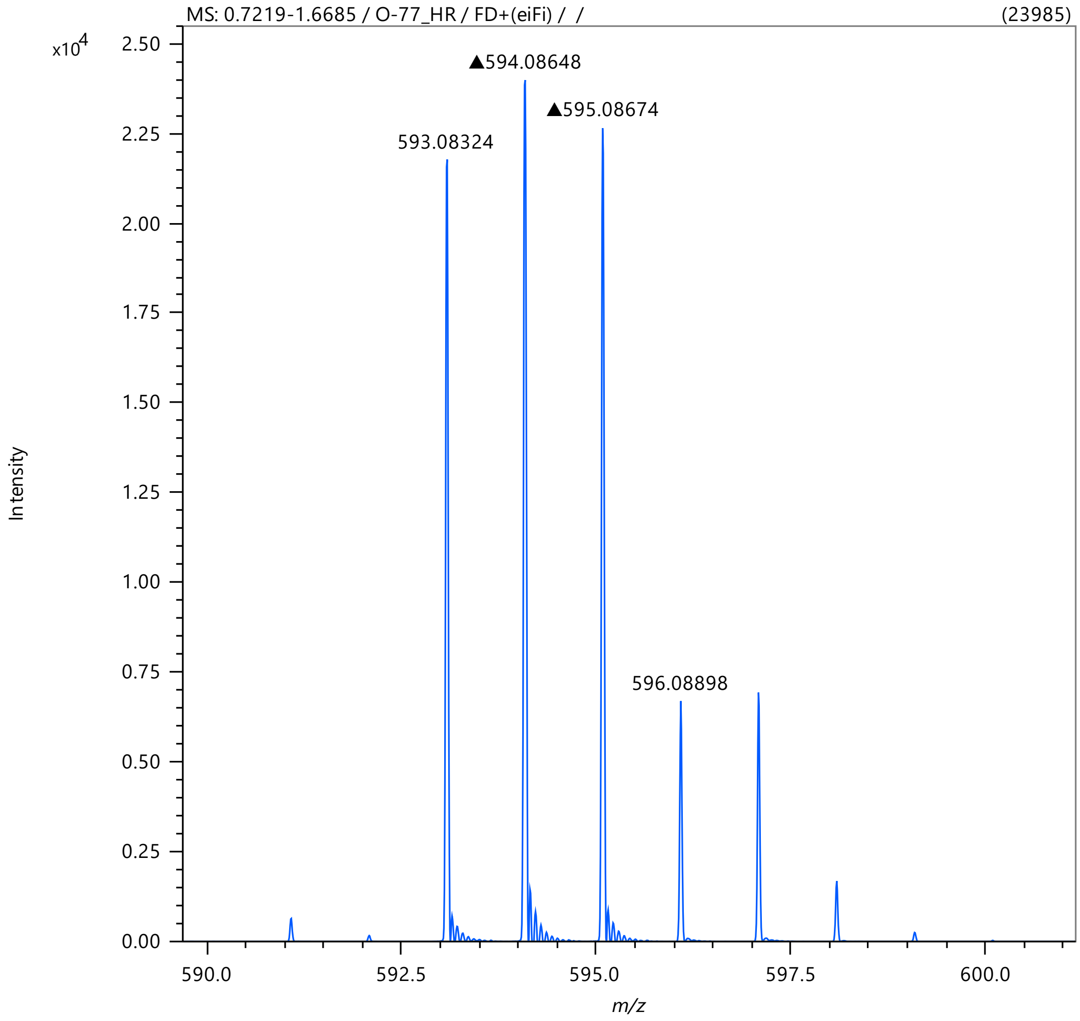

Figure S4. High-resolution mass spectrum (HRMS) of **LL-O**.

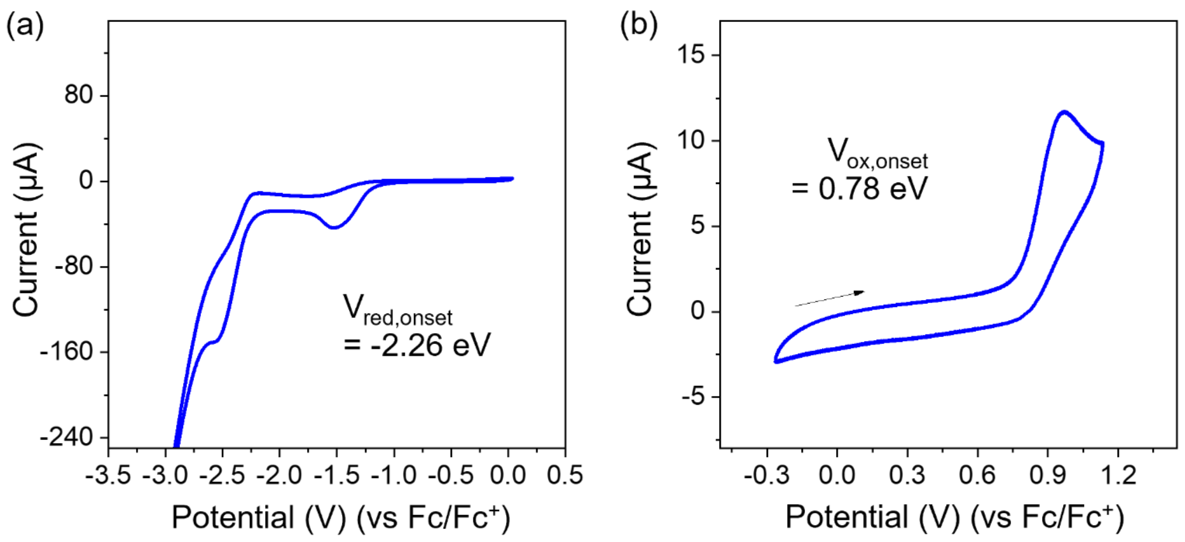

Figure S5. Consecutive cyclic voltammogram (CV) curves for **LL-O** showing (a) reduction and (b) oxidation (5 cycles, 0.5 mM in dichloromethane, scan rate = 100 mV/s).

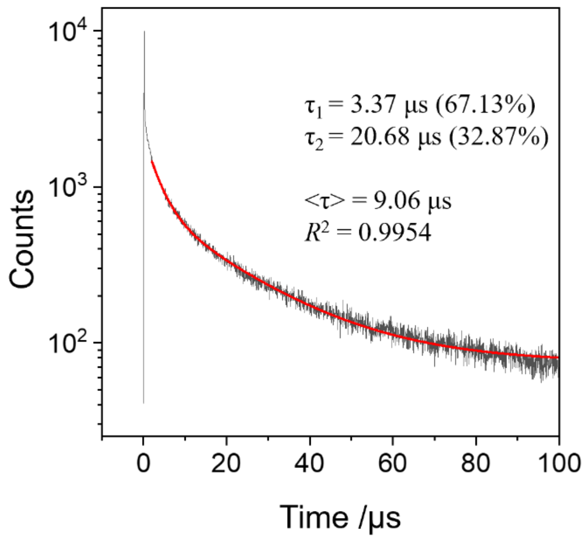

Figure S6. Emission decay curve obtained at each emission maximum for **LL-O** in the PMMA film states. The red-line corresponds to the double-exponential fitting curve for the experimental curve.

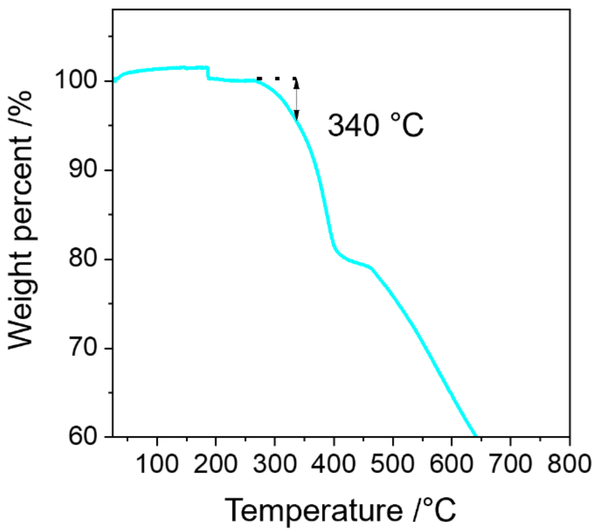

Figure S7. Thermogravimetric analysis curves of **LL-O**.

Theoretical calculation results for LL-O

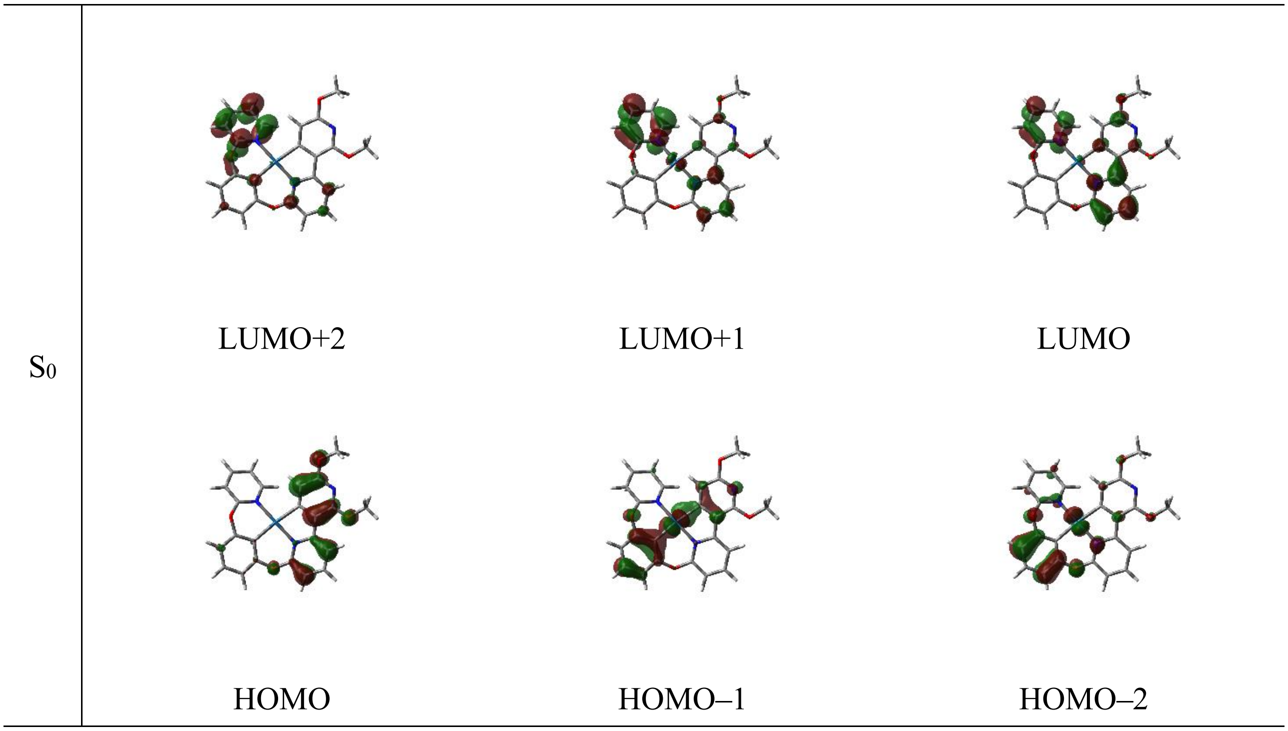

Figure S8. The selected frontier orbitals of **LL-O** from PBE0 calculations (Isovalue = 0.02 a.u.) at the ground state (S_0) optimized geometries in dichloromethane.

Table S1. Computed absorption wavelengths (λ_{calc}) and oscillator strengths ($f_{calc.}$) for **LL-O** in its ground state (S_0) optimized geometry in dichloromethane. The calculations were performed using the TD-PBE0 /GENCP method.

state	λ_{calc} (/nm)	f_{calc}	Major contribution		
1	352.50	0.0392	HOMO-1	→	LUMO (85.7%)
2	348.57	0.3049	HOMO	→	LUMO (85.7%)
3	327.04	0.0027	HOMO-1	→	LUMO+1 (80.43%)
4	315.98	0.0764	HOMO	→	LUMO+1 (59.9%)
5	313.09	0.0599	HOMO-2	→	LUMO (50.1%)

Table S2. Molecular orbital energies (E, eV) and molecular orbital distributions (%) of **LL-O** for the ground-state (S_0) optimized geometry in dichloromethane

	E	Pt	Methoxy pyridine	Phenyl	Terminal Pyridine	Pyridine
LUMO+3	-0.23	1.51	9.14	15.81	0.33	73.21
LUMO+2	-0.49	0.88	0.62	5.26	92.88	0.36
LUMO+1	-1.03	5.24	18.22	3.00	25.93	47.62
LUMO	-1.37	3.64	7.43	1.15	68.99	18.80
HOMO	-5.49	1.33	63.75	7.53	0.14	27.25
HOMO−1	-5.63	34.30	19.40	37.27	5.06	3.97
HOMO−2	-5.97	15.05	10.21	49.51	10.18	15.05
HOMO−3	-6.28	85.24	9.65	1.34	1.38	2.39

Table S3. Cartesian coordinates of the ground state (S_0) fully optimized geometry of **LL-O** from PBE0 calculations in dichloromethane (in Å)

Atom	X	Y	Z
C	-4.75012	-0.21632	1.023505
C	-3.47627	0.217712	0.672716
C	-2.475	-0.60809	0.170055
C	-2.83289	-1.9478	0.067175
C	-4.09425	-2.44645	0.391434
C	-5.05687	-1.56565	0.869818
H	-5.47614	0.492704	1.407735
H	-4.29874	-3.50634	0.280012
H	-6.04489	-1.931	1.13179
O	-1.96151	-2.92174	-0.39323
O	-3.2563	1.572162	0.925788
C	-2.46436	2.359359	0.191753
C	-2.90968	3.678063	0.038527
C	-2.17259	4.560204	-0.72341
H	-3.84341	3.956347	0.512184
C	-0.61324	2.799524	-1.12037
C	-1.00451	4.10197	-1.33719
H	-2.51055	5.58253	-0.85869
H	0.285631	2.394038	-1.56506
H	-0.40225	4.741792	-1.97107
C	-0.62375	-2.94016	-0.34527
C	-0.07865	-4.21606	-0.5235
C	1.293154	-4.35251	-0.5258
H	-0.75644	-5.0512	-0.6481
C	1.519322	-1.97789	-0.18856
C	2.099983	-3.23168	-0.35721
H	1.743189	-5.33127	-0.66034
H	3.175986	-3.31231	-0.36792
N	0.142432	-1.85258	-0.17622
N	-1.30405	1.934171	-0.33549
C	2.227575	-0.7153	-0.03949
C	3.620103	-0.57242	0.136849
C	1.424179	0.459631	-0.03152
C	2.073498	1.670983	0.188381
C	3.460268	1.683183	0.355981
H	1.546523	2.617334	0.252636
N	4.221474	0.595734	0.322095
O	4.401695	-1.6672	0.126075
O	4.047807	2.874459	0.561165
C	5.79899	-1.48312	0.323595
H	6.002237	-1.02624	1.295485
H	6.230318	-2.48348	0.279678
H	6.227451	-0.85195	-0.45915
C	5.457398	2.885364	0.742687
H	5.973312	2.491219	-0.13749
H	5.72495	3.931291	0.895883
H	5.748937	2.292115	1.614073
Pt	-0.57862	0.032519	-0.1194
